# Functional ultrastructure and cytochemistry of vitellogenesis and mature vitellocytes of the digenean *Cainocreadium labracis* (Dujardin, 1845), parasite of *Dicentrarchus labrax* (L., 1758)

**DOI:** 10.1007/s00436-018-6180-4

**Published:** 2018-12-26

**Authors:** Zdzisław Świderski, Hichem Kacem, John S. Mackiewicz, Jordi Miquel

**Affiliations:** 1 Witold Stefański Institute of Parasitology, Polish Academy of Sciences, 51/55 Twarda Street, 00-818 Warsaw, Poland; 2 Laboratoire de Biodiversité et Ecosystèmes Aquatiques, Département des Sciences de la Vie, Faculté des Sciences de Sfax, BP 1171, 3000 Sfax, Tunisia; 3 Department of Biological Sciences, University of New York at Albany, Albany, NY 12222 USA; 4 Secció de Parasitologia, Departament de Biologia, Sanitat i Medi Ambient, Facultat de Farmàcia i Ciències de l’Alimentació, Universitat de Barcelona, Av. Joan XXIII, sn, 08028 Barcelona, Spain; 5 Institut de Recerca de la Biodiversitat (IRBio), Universitat de Barcelona, Av. Diagonal, 645, 08028 Barcelona, Spain

**Keywords:** Digenea, *Cainocreadium labracis*, Vitellogenesis, Mature vitellocytes, Functional ultrastructure, TEM, Cytochemistry

## Abstract

Vitellogenesis and vitellocytes of *Cainocreadium labracis* were studied by transmission electron microscopy (TEM) and TEM cytochemistry. Four developmental stages were distinguished during vitellogenesis: (I) stem cell of high nucleo-cytoplasmic ratio; (II) early differentiation with chief activity focused on the beginning of protein synthesis and shell globule formation; (III) advanced differentiation with rapid intensification of protein synthesis, progressive fusion of single shell globules into large globule clusters, and formation of unsaturated lipid droplets surrounded by β-glycogen particles; and (IV) mature vitellocyte. Early vitellogenesis with vitellocyte maturation consists of: (1) increase in cell volume; (2) increased development of large, parallel cisternae of GER with production of proteinaceous granules; (3) development of small Golgi complexes that package granules; and (4) within vacuoles, progressive enlargement of proteinaceous granules into shell globule clusters formed during vitellogenesis. Three types of inclusions accumulate in large amounts in mature vitelline cells: (1) shell globule clusters, important component in the formation of egg shell; (2) numerous unsaturated lipid droplets. Though fewer, there are also diphasic droplets consisting of saturated and unsaturated lipids in the same droplet, and (3) a relatively small amount of β-glycogen particles, usually surround a few groups of lipid droplets. The β-glycogen and lipid droplets are nutritive reserves for embryogenesis. General pattern and functional ultrastructure of vitellogenesis greatly resemble those observed in some lower cestodes, such as bothriocephalideans and diphyllobothrideans. Variations and differences in the amount of lipids and of glycogen during vitellogenesis in lower cestodes and other trematodes are compared and discussed.

## Introduction

Vitellocytes of parasitic Platyhelminthes are a key element in the production of mature eggs containing invasive larvae (for a review, see Świderski and Xylander 2000). An interruption in vitellocyte formation results in immediate blockage of the infective egg production and thus an interruption of the parasite’s life cycle.

While much is known of the functional ultrastructure and cytochemistry of vitellogenesis and vitellocytes among parasitic Platyhelminthes, that include numerous cestode species from a wide range of hosts, similar TEM studies on trematodes appear somewhat neglected. Most work on trematodes has been focused on species of medical, veterinary, or economic importance, such as schistosomes or liver flukes, which have many intact vitellocytes (about 20–30) with the ovum in a large egg. Such studies include work on schistosomes by Erasmus et al. (1982), *Paragonimus ohirai* (Paragonimidae) by Fukuda et al. (1983), and *Fasciola hepatica* (Fasciolidae) by Björkman and Thorsell (1963), Thorsell et al. (1966), Irwin and Threadgold (1970), and Hanna (1976). Among the few studies on non-economically related trematode parasites include, e.g., papers on *Maritrema linguilla* (Microphallidae) by Hendow and James (1989), *Gorgoderina vitelliloba* (Gorgoderidae) by Irwin and Maguire (1979), and the aspidogastrean *Aspidogaster limacoides* by Levron et al. (2010).

As with cestodes, TEM data on vitellogenesis is desirable from a diverse sample of trematodes when considering how any character can be considered as useful for phylogenetic and evolutionary studies of the Platyhelminthes (Świderski and Xylander 2000; Świderski et al. 2009). Knowledge of vitellogenesis in diverse parasitic Platyhelminthes may also have an important applied aspect (Erasmus 1975; Mehlhorn et al. 1981; Shaw and Erasmus 1988) particularly with respect to measuring the effects of prospective antihelminthic drugs. Since degeneration of vitellocytes usually causes loss of egg production, it is possible to judge the effects of prospective ovicidal drugs by measuring their effects on the high metabolic rate of vitellocytes (Moczoń and Świderski 1983, 1992; Shaw and Erasmus 1988).

The aim of the present study is to describe the functional ultrastructure of vitellocytes and vitellogenesis in the trematode *Cainocreadium labracis* and compare it with data from other trematodes and some lower cestodes, chiefly parasites of fishes.

## Materials and methods

Live adult specimens of *C. labracis* (Dujardin, 1845) were collected in December 2015 from the digestive tract of the European seabass *Dicentrarchus labrax* (L., 1758) (Teleostei: Serranidae) from the Mediterranean Sea, off La Chebba (34° 14′ N, 11° 06′ E) (Tunisia).

Several worms were rinsed with a 0.9% NaCl solution and fixed in cold (4 °C) 2.5% glutaraldehyde in a 0.1-M sodium cacodylate buffer at pH 7.4 for a minimum of 2 h, rinsed in 0.1 M sodium cacodylate buffer at pH 7.4, post-fixed in cold (4 °C) 1% osmium tetroxide with 0.9% potassium ferricyanide in the same buffer for 1 h, rinsed in Milli-Q water (Millipore Gradient A10), dehydrated in an ethanol series and propylene oxide, embedded in Spurr’s resin, and polymerized at 60 °C for 72 h. Ultrathin sections (60–90 nm thick) were obtained using a Reichert-Jung Ultracut E ultramicrotome. Sections, placed on 200-mesh copper grids, were double-stained with uranyl acetate and lead citrate according to the Reynolds (1963) procedure and examined in a JEOL 1010 transmission electron microscope operated at an accelerating voltage of 80 kV, in the “Centres Científics i Tecnològics” of the University of Barcelona (CCiTUB).

Sections placed on gold grids were treated according to the Thiéry (1967) test to reveal the presence of glycogen. Thus, they were treated in periodic acid (PA), thiocarbohydrazide (TCH), and silver proteinate (SP) as follows: 30 min in 10% PA, rinsed in Milli-Q water; 24 h in TCH, rinsed in acetic solutions and Milli-Q water; and 30 min in 1% SP in the dark and rinsed in Milli-Q water. Sections were examined in a JEOL 1010 transmission electron microscope in the CCiTUB.

## Results

### General topography of the vitelline system

**Fig. 1:**
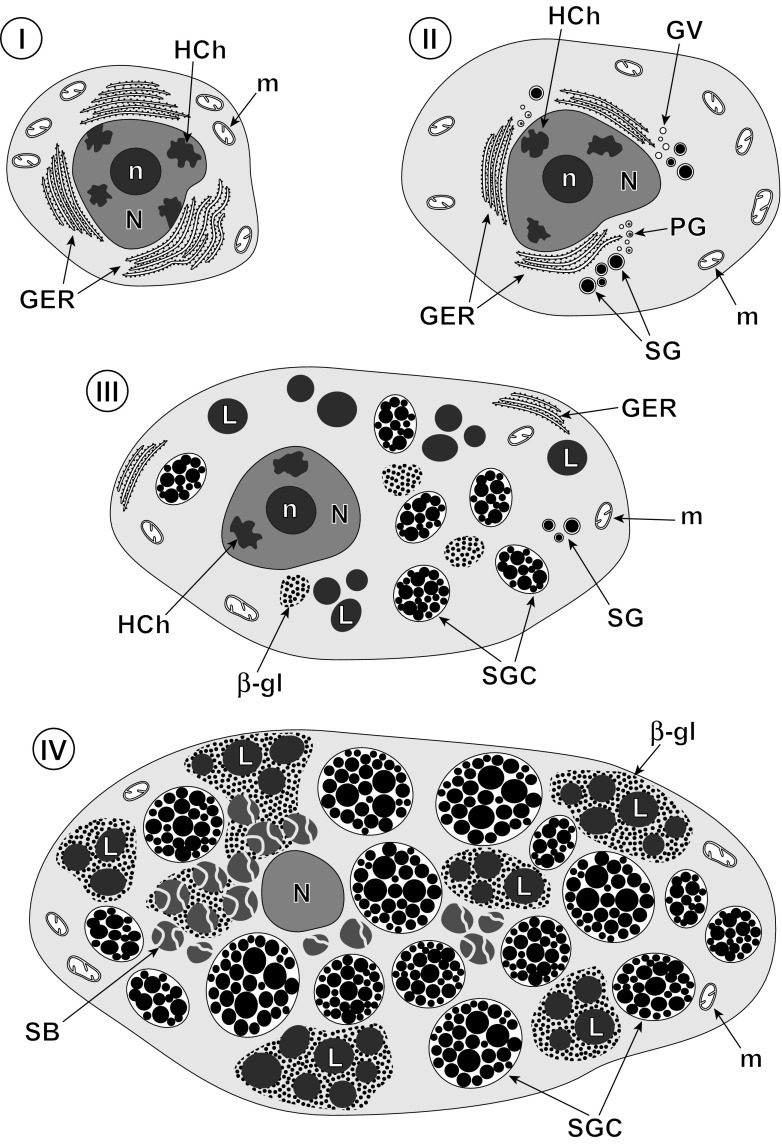
Diagram of four developmental stages of vitellogenesis in digenetic trematode *Cainocreadium labracis*. Note four consecutive stages of vitellogenesis reflecting vitellocyte cytodifferentiation and maturation: (stage I) a stem cell stage of the gonial type, (stage II) an early differentiation stage, (stage III) an advanced stage of maturation, and (stage IV) mature vitellocyte. The early stages (I and II) are predominantly at the periphery of the follicles, whereas the more advanced stage of maturation (stage III) and mature vitellocytes (stage IV) are localized mainly in their central region. *I–IV* consecutive stages of vitellogenesis, *I* stem cell of gonial type, *II* early and *III* advanced stages of vitellocyte differentiation, *IV* mature vitellocyte, *β-gl* beta-glycogen, *GER* granular endoplasmic reticulum, *GV* Golgi vesicles, *HCh* heterochromatin islands, *L* lipid droplets, *m* mitochondria, *N* nucleus, *n* nucleolus, *PG* proteinaceus granules, *SB* spherical bodies, *SG* shell globules, *SGC* shell globule clusters

**Fig. 2:**
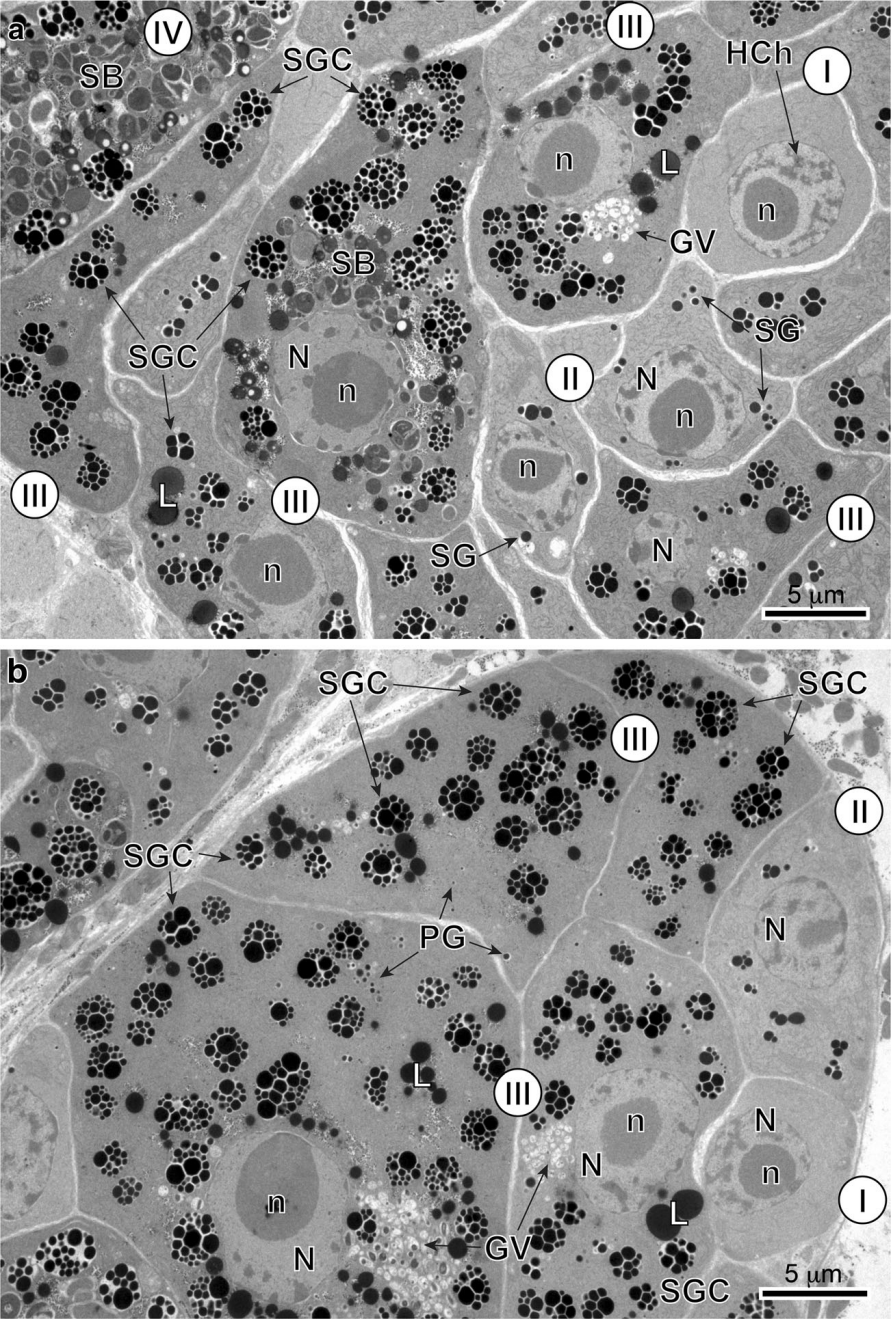
**a** and **b** General topography of the vitelline follicle showing the localization of vitellogenesis developmental stages I–III within the follicle. Note the peripheral position of stem cell of gonial type and central localization of advanced stages of vitellogenesis reflecting gradient of cytodifferentiation. *I* stem cell of gonial type, *II* early stage of vitellocyte differentiation, *III* advanced stage of differentiation and *IV* mature vitellocyte, *GV* Golgi vesicles, *HCh* heterochromatin islands, *L* lipid droplets, *N* nucleus, *n* nucleolus, *PG* proteinaceus granules, *SB* spherical bodies, *SG* shell globules, *SGC* shell globule clusters

The vitellaria or vitelline glands of the digenetic trematode *C. labracis* are follicular as described by Bartoli et al. (1989) as follows: The forebody lateral fields of vitellaria are restricted to the dorsal plane, sometimes fusing medially, whereas in the hindbody lateral fields occur dorsally and ventrally. Vitelline fields fuse in the post-testicular zone and extend to the level between the intestinal bifurcation and mid-pharynx in the anterior direction (see Bartoli et al. 1989). The size of vitelline follicles can vary from 15 to 40 μm. Vitellocytes at different stages of maturation are close to each other (Figs. 1 stages I–IV and 2 a, b). No interstitial or nurse cells were observed. Although vitellogenesis is a continuous process, we follow the system that divides the process into four discrete developmental stages (Figs. 1 stages I–IV; 2a, b; 3a, b; 4a, b; and 5a–c) described previously by Irwin and Threadgold (1970) in digeneans and by Świderski and Mokhtar (1974) in cestodes. Terminology is that of Świderski and Xylander (2000). These four stages of vitellogenesis in *C. labracis* are illustrated diagrammatically on Fig. 1. We distinguished: (stage I) a stem cell of gonial type, (stage II) early differentiation, (stage III) advanced differentiation, and (stage IV) mature vitellocyte. Stages I and II are chiefly at the follicle edge, while stages III and IV are usually in the central part of the cell (Fig. 2a, b). As a whole, vitellogenesis and vitellocytes are very similar to that described from *Maritrema feliui* by Świderski et al. (2011a).

**Fig. 3:**
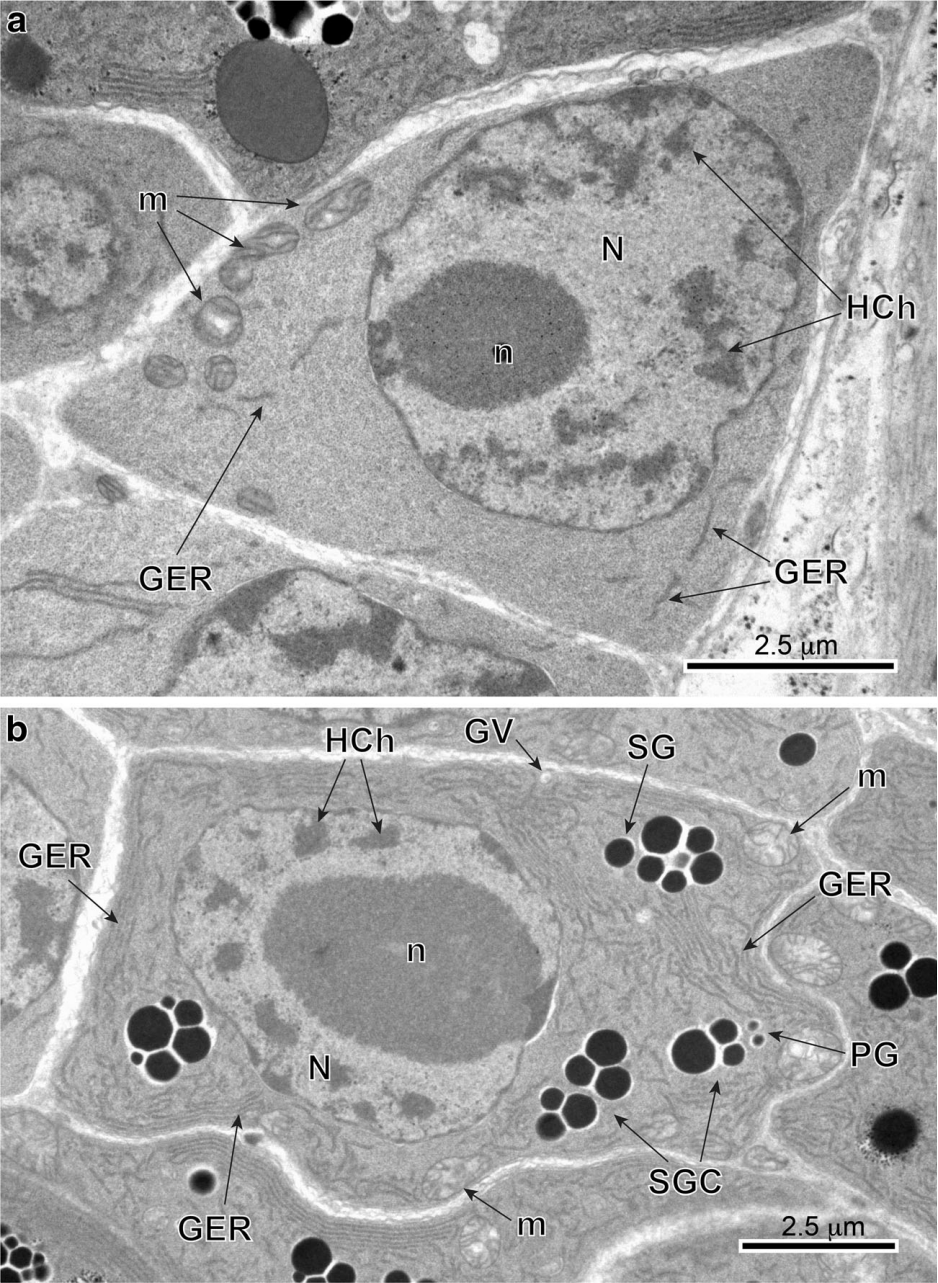
**a** and **b** High-power TEM-micrographs illustrating ultrastructural details of a stem cell of the gonial type (stage I) (**a**) and an earliest stage (II) of vitellocyte cytodifferentiation (**b**). Compare differences in the ultrastructure of their cell organelles and inclusions. Note: (1) very large nuclei (*N*) contain prominent spherical nucleoli (*n*) and numerous large islands of heterochromatin (*HCh*) adjacent to the nuclear envelope or randomly dispersed in the nucleoplasm, and (2) a very high nucleo-cytoplasmic ratio. The granular cytoplasm contains extended profiles of granular endoplasmic reticulum (*GER*) adjacent to Golgi complexes, with a few membrane-bounded vesicles containing dense material of shell globule primordia and several small shell globule clusters (*SGC*) at stage II of early vitellocyte differentiation. *GV* Golgi vesicles, *m* mitochondria, *PG* proteinaceus granules, *SG* shell globules

**Fig. 4:**
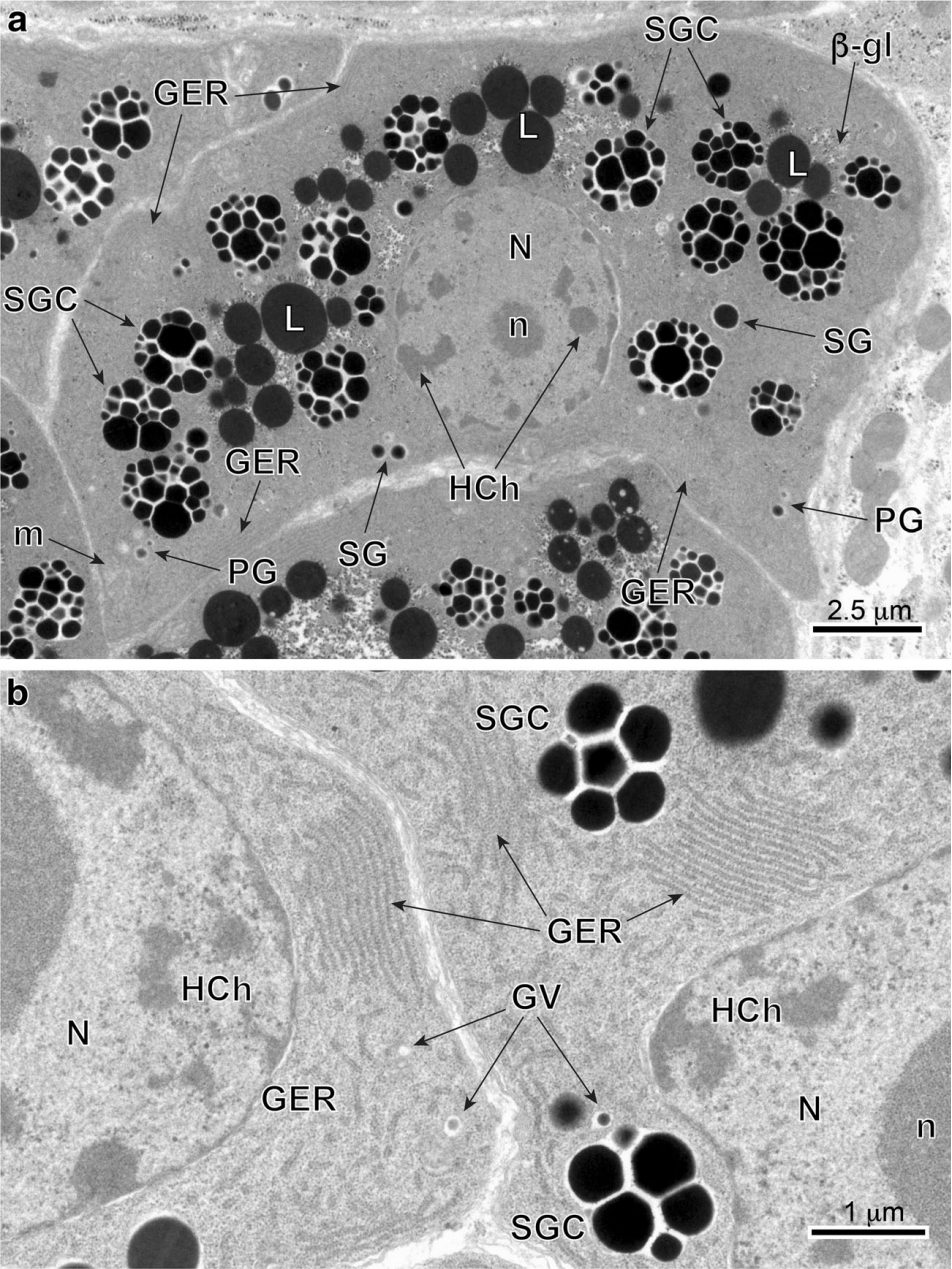
**a** and **b** TEM micrographs illustrating advanced stages of vitellocyte cytodifferentiation (stage III). Note: (1) parts of large nuclei (*N*), with several heterochromatin islands, randomly dispersed in the nucleoplasm; (2) extended areas of well-developed, parallel cisternae of GER; (3) numerous cell inclusions, namely shell globules (*SG*) and shell globule clusters (*SGC*), several groups of unsaturated lipid droplets (*L*) surrounded by accumulations of β-glycogen particles (*β-gl*) and a few spherical bodies. Note the large vitellocyte, which in spite of a very heavy accumulation of numerous shell globule clusters, still contains a large nucleus (*N*) with prominent nucleolus (*n*) and well-developed cytoplasmic organelles, such as extended GER cisternae, Golgi complexes, and numerous mitochondria (*m*), which may confirm active protein synthesis. *GER* granular endoplasmic reticulum, *GV* Golgi vesicles, *HCh* heterochromatin islands, *PG* proteinaceus granules

**Fig. 5:**
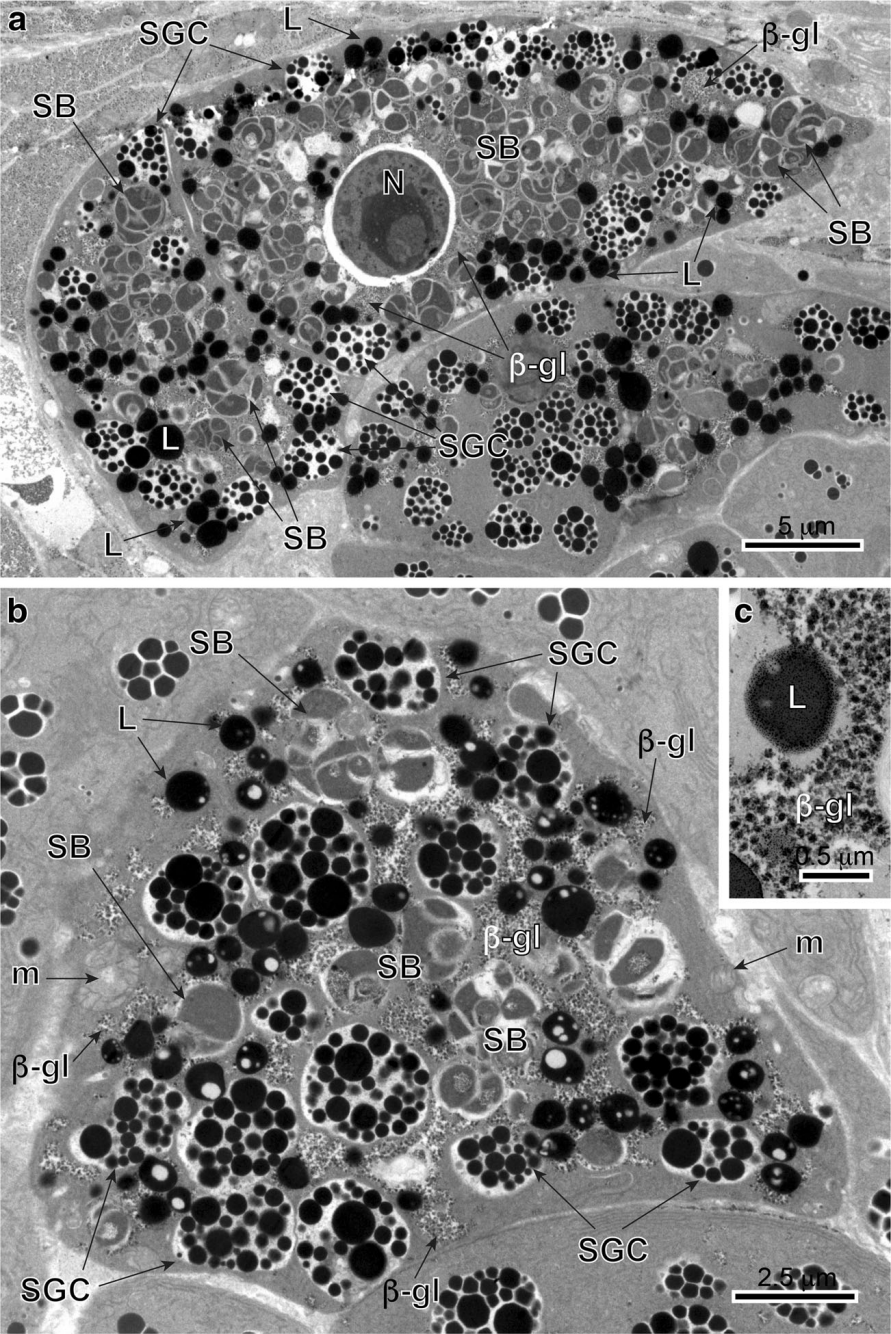
**a**-**c** TEM-micrographs illustrating mature vitellocytes (stage IV). In **a**, note the presence of degenerating nucleus (*N*) in a mature vitellocyte. The cytoplasm is completely filled by large amounts of shell globule clusters (*SGC*), unsaturated lipid droplets (*L*), spherical bodies (*SB*), and β-glycogen particles (*β-gl*). Glycogen particles are mainly surrounding shell globule clusters and lipid droplets (**b** and **c**). *m* mitochondria

### Ultrastructure of consecutive stages

#### Gonial-type stem cell

Gonial cells about 6–7 μm in diameter have a high nucleo-cytoplasmic ratio and are usually at the follicle periphery (Figs. 2b and 3a). The thin layer of cytoplasm has a large concentration of free ribosomes, few mitochondria, and short profiles of granular endoplasmic reticulum (Fig. 3a). Prominent, spherical nucleoli characterize the large nuclei. The nucleoplasm has many randomly dispersed islands of heterochromatin (Fig. 3a).

#### Early and advanced differentiation stages: protein synthesis and shell globule formation

**Fig. 6:**
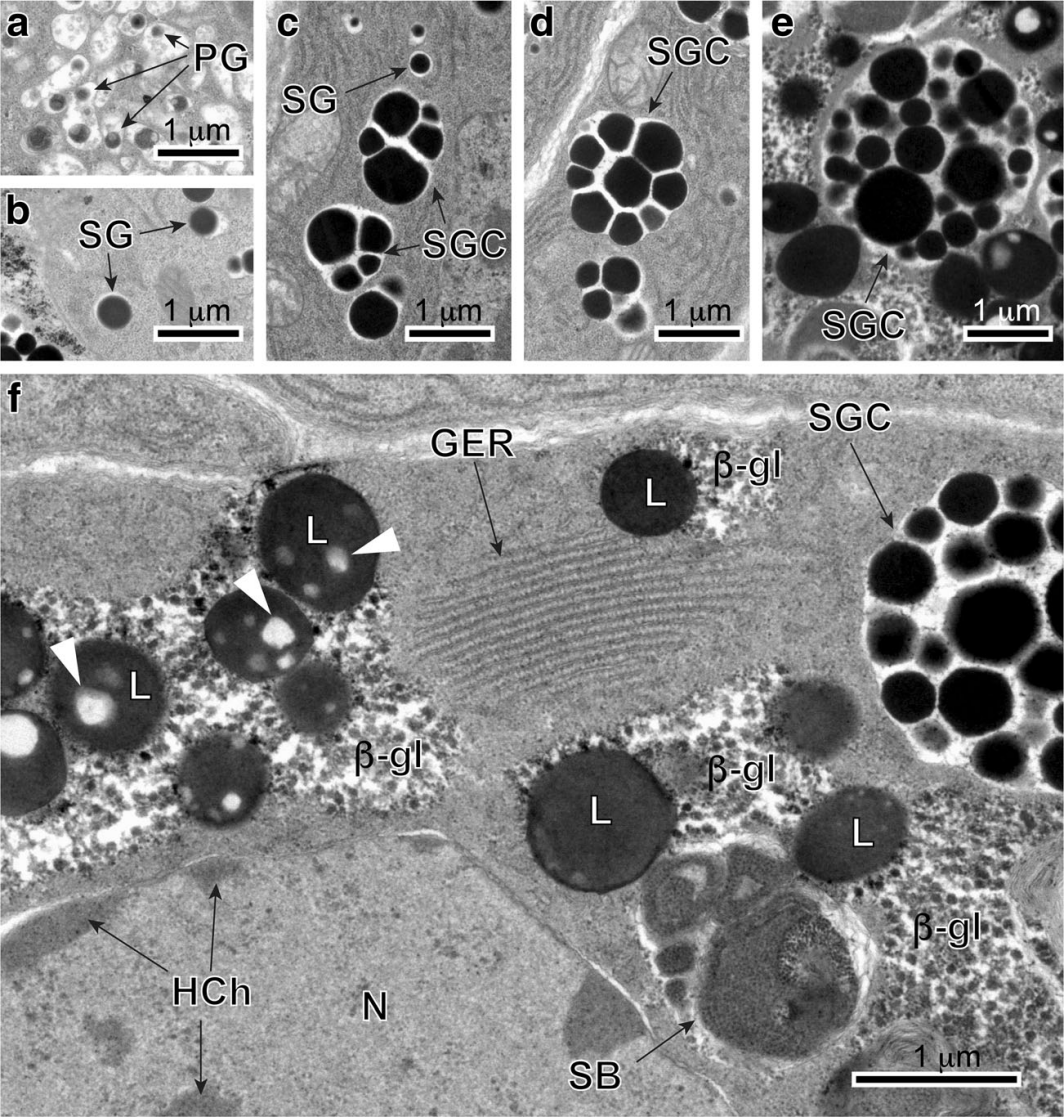
**a**-**f** High-power TEM micrographs illustrating transformation of proteinaceus granules into shell globule clusters (**a**–**e**) and some details of mature vitellocyte (**f**). Note several large diphasic, unsaturated lipid droplets (*L*) with small circular areas of low electron density resembling that of the osmiophobic saturated lipids (*white arrowheads*). *β-gl* beta-glycogen, *GER* granular endoplasmic reticulum, *HCh* heterochromatin islands, *N* nucleus, *PG* proteinaceus granules, *SB* spherical bodies, *SG* shell globules, *SGC* shell globule clusters

Early stage II (Figs. 1 and 3b) and stage III (Figs. 1 and 4a) cells characteristically increase in size rapidly, up to approximately 10–12 μm in diameter. Their nuclei show large, spherical nucleoli and numerous heterochromatin islands. At the same time, the cytoplasm shows increase in number of mitochondria, formation of numerous large, parallel profiles of granular endoplasmic reticulum (GER), and adjacent to them small vesicles originating from Golgi complexes. The smallest individual proteinaceous shell granules are synthesized in the GER and packaged as small granules in membrane-bounded Golgi vesicles. It appears that there is further growth and differentiation of the granules, increasing their size and forming shell globules (Figs. 3b; 4a, b; and 6a–c). The fusion of membranes of several proteinaceous shell globules results in formation of shell globule clusters (Figs 4a, b; 5a, b; and 6c–e). The size of shell globule clusters and the number of electron-dense islands or sub-units in the clusters increase progressively into mature vitellocytes (compare Figs. 4a; 5a, b; and 6c–e). A cluster is made up of numerous loosely packed electron-dense globules of various sizes embedded in a moderately electron-dense matrix (Figs 5a, b and 6e). Transformation of proteinaceous granules into shell globule clusters resulting from individual shell globule fusion is illustrated in Fig. 6a–e. This progressive advanced stage of maturation is apparently completed when a large number of shell globule clusters is formed within the vitellocyte cytoplasm.

#### Mature vitellocyte stage

**Fig. 7:**
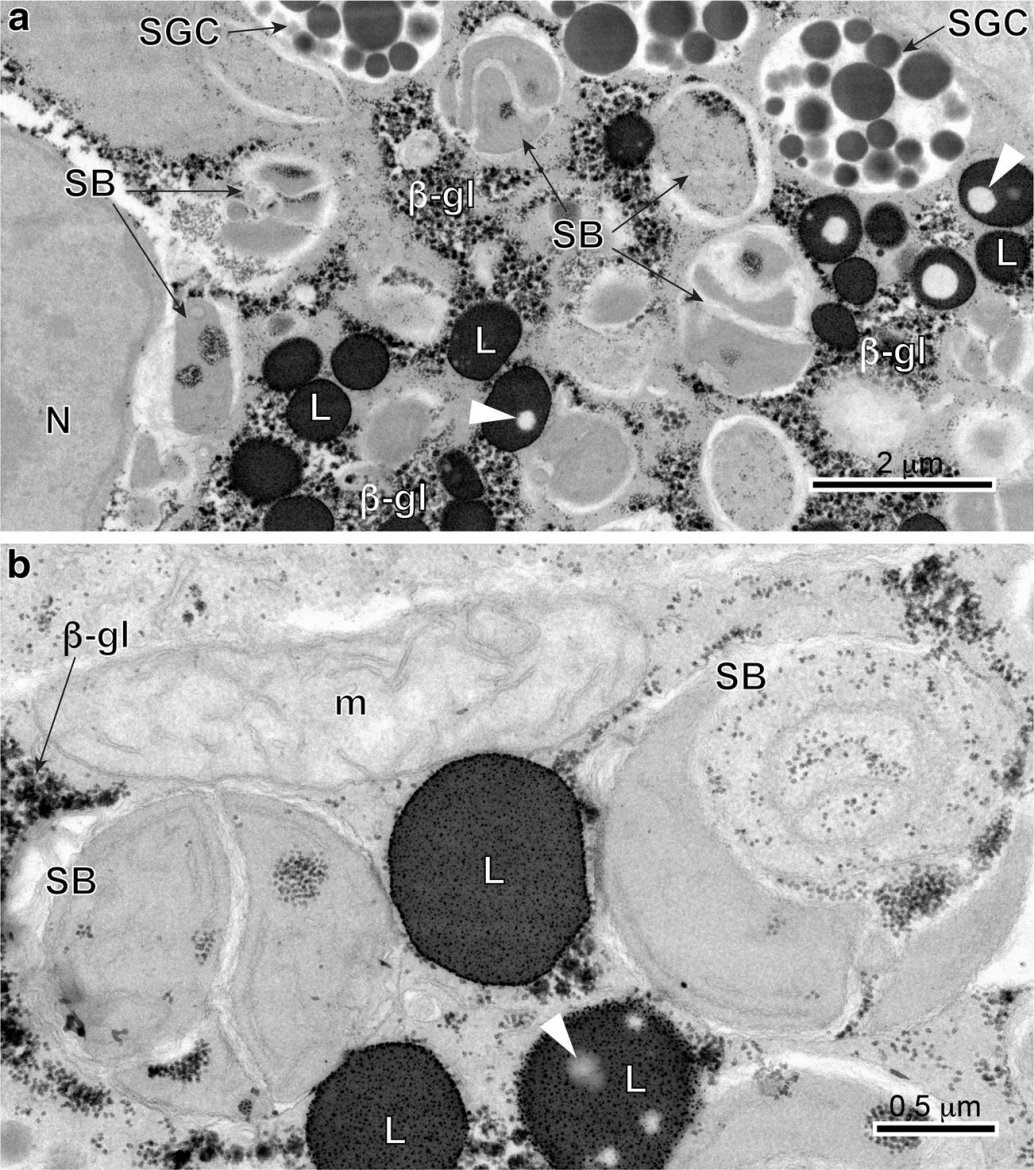
**a** and **b** High-power TEM-micrographs showing positive reaction for glycogen after the Thiéry test and illustrating ultrastructural details of mature vitellocytes. Note a heavy accumulation of four types of cell inclusions: shell globule clusters (*SGC*), diphasic unsaturated lipid droplets (*L*) with low electron-dense islands (*white arrowheads*), β-glycogen particles (*β-gl*), and several spherical bodies (*SB*). *m* mitochondria,* N* nucleus

Stage IV or mature vitellocyte (see: Figs. 1 and 5a, b), measuring about 15 μm in length by about 18 μm in width, is characterized by a high accumulation of four types of inclusions: (1) heterogeneous shell globule clusters, (2) numerous unsaturated lipid droplets of two kinds, (3) small accumulations of β-glycogen particles surrounding the lipid droplets, and (4) a few spherical bodies (Figs. 5a, b; 6f; and 7a, b). Spherical bodies resemble two or three mitochondria grouped together, which are surrounded by a common plasma membrane. The degenerating nuclei were found in some mature vitellocytes (Fig. 5a); however, they were not found in other vitellocytes (Fig. 5b). The lipid droplets in the mature vitellocyte of *C. labracis* appear to be of two kinds: one is completely dense, highly osmiophilic, of unsaturated lipids (Figs. 5b; 6f; and 7a, b) and the other is a diphasic droplet that is dense, highly osmiophilic, and containing from one to five round osmiophobic inclusions of various sizes (Figs. 5b, c; 6f; and 7a, b). Some of the inclusions are quite large, giving the droplet the appearance of a donut (Fig. 7a). We did not observe any droplets that were completely of low electron density because of only saturated lipid.

## Discussion

The ultrastructural characteristics of vitellogenesis in *C. labracis* are essentially similar to those described previously in different species of digenean trematodes, i.e., *Fasciola hepatica* (see Irwin and Threadgold 1970), schistosomes (Erasmus 1975; Erasmus et al. 1982), and *Maritrema* spp. (Hendow and James 1989; Świderski et al. 2011a). To some extent, they resemble also those reported in some lower cestodes, i.e., bothriocephalideans (Świderski and Mokhtar 1974) or diphyllobothrideans (Yoneva et al. 2014, 2015). The caryophyllideans, however, are exceptional in this respect among all other Platyhelmithes. They show an entirely different type of vitellogenesis, characterized by a large amount of not only cytoplasmic glycogen but also a unique, large accumulation of so-called “nuclear glycogen,” never observed in other normally developing cells, except in human liver cancer cases (for a review, see Świderski and Mackiewicz 1976).

More recent papers on vitellogenesis and vitellocytes of digeneans concern *Crepidostomum metoecus* (Allocreadiidae), *Azygia lucii* (Azygiidae), *Aphallus tubarium* and *Metadema depressa* (Cyptogonimidae), *Phyllodistomum angulatum* (Gorgoderidae), *Plagiorchis elegans* (Plagiorchiidae), or *Brandesia turgida* (Pleurogenidae) (see Table 1 for a comparative analysis of characteristics of mature vitellocytes).

**Table 1:** General characteristics of mature vitellocytes in some digeneans

Families and species	General characteristics of mature vitellocytes											References
	*N*	SGC	L	gl	GER	m	MW (=GERb?)	YG (=GERb?)	cGER (=GERb?)	DI	SB	
Allocreadiidae												
*Crepidostomum metoecus*	+	+	S	β	+	+						Greani et al. (2016)
Azygiidae												
*Azygia lucii*	+	+	S	+	+							Poddubnaya et al. (2012)
Cryptogonimidae												
*Aphallus tubarium*	+	+	S	β	+	+						Greani et al. (2012a)
*Metadema depressa*	+	+	S	β	+	+						Greani et al. (2012b)
Diplostomidae												
*Pharyngostomoides procyonis*	deg.	+	S	β		+	+					Grant et al. (1977)
Fasciolidae												
*Fasciola hepatica*	+	+		α, β	+			+				Irwin and Threadgold (1970)
Gorgoderidae												
*Gorgoderina vitelliloba*	+	+	S		+	+						Irwin and Maguire (1979)
*Phyllodistomum angulatum*	+	+	S		+							Poddubnaya et al. (2012)
Microphallidae												
*Maritrema feliui*	–	+	S	β								Świderski et al. (2011)
*M. linguilla*	deg./−	+	S	–		+		–				Hendow and James (1989)
Plagiorchiidae												
*Plagiorchis elegans*	+	+	S	α, β	+				+			Greani et al. (2014)
Pleurogenidae												
*Brandesia turgida*	+	+	S	–	+	+		+				Poddubnaya et al. (2013)
Schistosomatidae												
*Schistosoma haematobium*, *S. japonicum*, *S. mansoni*, *S. mattheei*	+	+	S	α^a^						+^b^		Erasmus et al. (1982)
Opecoelidae												
*Cainocreadium labracis*	deg./−	+	US	β		+					+	Present study

*cGER* dense circular GER, *deg.* degenerating, *DI* dense inclusions, *GER* granular endoplasmic reticulum, *GERb* GER bodies, *gl* glycogen, *L* lipid droplets, *m* mitochondria, *MW* membranous whorls, *N* nucleus, *S* saturated, *SB* spherical bodies, *SGC* shell globule clusters, *US* unsaturated, *YG* yolk globules, *±* presence/absence

^a^In all species, except in *S. japonicum*

^b^Only in *S. japonicum*

Vitelline cells of trematodes and cestodes play two very important functions: (1) formation of a hard, dense, and resistant egg shell, and (2) storage and supplying of nutritive reserves for the developing embryos. It, therefore, is essential for production of mature eggs containing invasive larvae (for a review, see Świderski and Xylander 2000).

Egg shell formation takes place in the ootype and results from the combined action of shell globules of vitelline cells and Mehlis’ gland secretion (Smyth and Clegg 1959). The Mehlis’ gland PAS-positive secretion acts on the groups of vitelline cells surrounding each fertilized oocyte passing through the ootype, causing release of all of the shell globules that fuse together to form a thick, electron-dense egg shell. Ultrastructure and histochemistry of egg shell formation have been described in different species of digeneans (Eklu-Natey et al. 1982a, 1982b; Świderski 1984, 1985, 1994).

An important aspect of vitellogenesis in trematodes concerns the nature of the glycogen reserves. Much is known of the ultrastructural aspects of glycogen reserves in mature vitellocytes of *F. hepatica*, and medically important species of schistosomes; for details, see the comparative Table 1. The amount of glycogen reserves shows great differences in these two digenean taxa. In *F. hepatica*, the reserves are always very heavy (Björkman and Thorsell 1963; Thorsell et al. 1966; Irwin and Threadgold 1970; Hanna 1976). This glycogen occurs as both α-glycogen rosettes for a long storage, and single β-glycogen particles for rapid, immediate utilization (see Świderski and Mackiewicz 1976; Świderski et al. 2004, 2009). On the other hand, in schistosomes (Erasmus et al. 1982; Świderski 1984, 1985, 1994), some lower cestodes (Świderski and Xylander 2000), and in *C. labracis*, the glycogen is only in very small accumulations of single β-glycogen particles for a short storage.

In schistosomes, this condition may be explained by the fact that miracidia develop in eggs that are in the host tissue (Świderski 1984, 1985, 1988, 1994). Since these miracidia have been shown to utilize exogenous metabolites from the host tissue (Sternholm and Warren 1974; Kawanaka et al. 1983), little nutrient reserves are required from vitellogenesis, with most vitelline metabolites being used for shell formation. Indeed, it may be that the schistosome egg itself is parasitic, as suggested by Tinsley (1983).

With regard to *C. labracis*, the very limited amount of nutritive reserves in their vitelline cells may probably be explained also by its life cycle. As outlined by Maillard (1976), the three-host life cycle can be briefly summarized as follows. The adult stage parasitizes the mid-intestine of the definitive host, sea bass *Dicentrarchus labrax*, and also other teleosts. Miracidia that hatch from eggs are infecting the first intermediate host, gastropods, mainly *Gibbula adansoni*. The cotylicerc cercariae leave the mollusk and infect the second intermediate host, small benthic teleosts, mainly gobies (Gobiidae), where they form encysted metacercariae. The metacercaria produces paralysis of gobies’ fins and disorders in the normal function of the eyes, thus facilitating the predatory action of the sea bass acting as a final host. The metacercaria then completes its development and transforms into an adult stage in the intestine of the sea bass.

Comparison of the life cycle of *C. labracis* indicates that, as with *Schistosoma* spp., the protective role of vitelline shell globules in the egg shell formation appears more important than the nutritive role of the small amount of β-glycogen particles and unsaturated lipid droplets. The nutrition of the developing embryos is probably supplied by intrauterine embryogenesis. Unfortunately, there is no information on the type of embryogenesis in this species.

Vitellogenesis like that described in our study has been observed in lower cestodes as follows: in gyrocotylids by Xylander (1987), amphilinids by Xylander (1988), caryophyllidids by Bruňanská et al. (2012, 2013a, 2013b), bothriocephalids by Świderski and Mokhtar (1974) and Levron et al. (2007), and spathebothriids by Bruňanská et al. (2005) and Poddubnaya et al. (2005, 2006). Common to these cestodes is a similar egg type, with a thin shell and operculated, resembling the eggs of most trematodes. These cestodes also have vitellocytes with heavy accumulations of shell globule clusters and lipid droplets, though generally less glycogen. Caryophyllidea, an order of lower monozoic cestodes of freshwater fishes, differs greatly from *C. labracis* as well as from Amphilinidea and Gyrocotylidea by having a very large amount of α-glycogen rosettes and β-glycogen particles in the cytoplasm, with dense concentrations in the nucleoplasm (Mackiewicz 1968; Świderski and Mackiewicz 1976; Świderski et al. 2004, 2009; Bruňanská et al. 2009).

Little is known of spherical bodies that are described for the first time in this study. As far as we know, they have never been reported previously in vitellocytes of other Platyhelminthes.

Unlike glycogen, the functional significance of lipids in the vitellocytes of *C. labracis*, or other trematodes, is less well understood. As highly complex and diverse organic compounds, lipids may function in many ways, including energy source, component of biological membranes, and regulator of cellular activity. According to Smyth and Halton (1983), lipids are generally considered an important energy reserve, though this may not be true for all trematodes. The apparent absence of lipid droplets of osmiophobic saturated lipid droplets in *C. labracis*, as found in *M. feliui*, for example (see Fig. 4 of Świderski et al. 2011a), is puzzling. The significance of diphasic droplets consisting of saturated (osmiophobic) and unsaturated (osmiophilic) lipids in the same droplet is unknown. Are they stages of lipid synthesis hitherto seldom observed? Do they represent another kind of lipid droplet made up of a mix of unsaturated and saturated lipids rather than droplets of individual types of lipid? Or is this just another example of the “remarkable diversity” recognized by Conn et al. (2018) as present even in closely related trematodes? We do not know. We are not aware of similar diphasic lipid droplets in any other trematode. Concerning cestodes, however, diphasic lipid droplets have been previously found in the vitellocytes of *Echinobothrium euterpes* (Diphyllidea) (see Świderski et al. 2011b). On the other hand, they have never been observed in other cestodes such as trypanorhynchids (see Świderski et al. 2012) or proteocephalideans (for a review, see Świderski and Xylander 2000). The fact that the saturated islands within the droplet are of different size and number suggests that there may be a conversion of unsaturated lipid to the saturated state. However, this does not explain why no droplets of only saturated lipid were observed. Clearly, much remains to be learned of lipid metabolism in diverse taxa of parasitic Platyhelminthes.
